# 

**Published:** 1872-01

**Authors:** 


### ANNUAL MEETING.

The annual meeting of the Mississippi Valley Dental Society will be held in the Hall of the Ohio Dental College, on Wednesday, the 6th of March, at 10 o'clock A. M., when it is confidently expected that there will be a large number of the membership and of the profession present. This is the oldest Dental Society in existence, and one of the most active and energetic. It has been the projector of many good works in the profession. A cordial invitation is extended to all members of the profession to be present. The order of business will be in the February number.

### CONTENTS.

COMMUNICATIONS.

### The Dental Register,

### OF THE WEST.

A Monthly Magazine, devoted entirely to the interest of the Dental Profession.

**Terms $3.00 per Year. Single Nos. 25 Cents.**

EDITED BY PROF. J. TAFT,

And Published by SPENCER & MOORE of the Ohio Dental Depot.

The volume commences in January and closes in December.

The Register being issued monthly is always fresh, therefore indispensable to the practitioner who desires to keep posted up with the new inventions and improvements which are daily developed. Neither expense nor effort will be spared to give value and variety to its contents. Articles will be illustrated with well executed engravings whenever they will add to the interest or assist in explanation.

Its extensive circulation and frequency of issue makes it one of the BEST DENTAL ADVERTISING MEDIUMS in the United States.

RATES OF ADVERTISING.

One page, one year, $50. Half page, one year, $30. Quarter page, one year, $20.

All advertising bills payable in advance, or at furthest after the issue of the first number containing the advertisement. All transient advertisements to be paid for in advance.

☞CONTRIBUTIONS SOLICITED.

Exclusively Editorial Communications should be addressed to

**Dr. J. TAFT, Editor.**

The latest number of the Register may be ordered with or without other goods at any time, and all letters should be addressed to DENTAL REGISTER,

Or **SPENCER & MOORE,**

***Room No. 1 Pike's Opera House, Cincinnati, O.***

